# A case of intravenous iron administration resulting in cerebral edema expansion

**DOI:** 10.1186/s12883-023-03258-8

**Published:** 2023-05-30

**Authors:** Jonathan Espinosa, Umair Rehman, Firas Kaddouh

**Affiliations:** 1 Department of Neurosurgery, University of Texas Health Science Center San Antonio, San Antonio, TX USA; 2 Department of Neurology, University of Arizona College of Medicine, Tucson, AZ USA

**Keywords:** Cerebral edema, Intracerebral hemorrhage, Traumatic brain injury, Intravenous iron, Perihematomal edema, Case report

## Abstract

**Background:**

Iron plays an important role in the development of perihematomal edema (PHE) in the setting of intracerebral hemorrhage (ICH). Cerebral iron is increased via direct hemoglobin release in ICH, and several studies have investigated the use of iron-chelating agents to mitigate its toxicity. However, the effect of systemic iron administration, corroborating the reverse concept, has never been investigated or reported clinically. We report the first case of systemic iron administration in the setting of hemorrhagic traumatic brain injury (TBI).

**Case presentation:**

A 46-year-old woman was admitted to the hospital with acute moderate-to-severe TBI. Her head computed tomography (CT) scan showed bifrontal hemorrhagic contusions with mild PHE. She was started on hypertonic saline 3% continuous infusion and her condition remained stable initially. She was found to be anemic and was given intravenous iron sucrose. Shortly after iron administration, her mental status declined, and left pupil became dilated and sluggish. Repeat CT demonstrated significantly worsening PHE. This prompted maximum hyperosmolar therapy and external ventricular drain (EVD) placement which both were weaned off slowly due to liable ICPs. She was discharged home after a 25-day hospital stay.

**Conclusions:**

We believe this is the first report of exacerbating PHE accompanied by clinical decline after intravenous iron administration in the setting of acute hemorrhagic brain contusions. Though the effects of systemic iron administration on brain edema and the treatments targeting cerebral iron are poorly understood, the administration of systemic iron in acute TBI seems to be detrimental. More research is needed to address iron toxicity in TBI. Our case adds to the growing evidence for such a pathway in the treatment of ICH and TBI.

## Background

Iron has an important role in the development of perihematomal edema (PHE) associated with intracerebral hemorrhage (ICH). Though the topic is not well understood, free iron is considered highly toxic to neural tissue due to its ability to form free radicals and cause significant oxidative stress. In the setting of ICH, free iron is released from the hematoma via lysis of red blood cells [1]. The acute increase in local iron at the time of hemorrhage is a likely contributor to the formation of cerebral edema leading to secondary brain injury. Iron accumulation also correlates with brain atrophy development in the late stage of ICH. Thus, it is plausible that accumulation of iron within the brain may be related to the clinical outcome of ICH [2].

Herein, we report a case of acute traumatic brain injury (TBI), with bifrontal hemorrhagic contusions, in which the patient experienced a decline in neurological status with exponentially increased perihematomal brain edema concurrent with the administration of intravenous (IV) iron. This case potentially demonstrates the toxic effect that elemental iron has on the brain and calls to caution and avoidance of iron administration in the setting of acute brain injury. To our knowledge, this is the first reported case to show such effect as a consequence of systemic IV iron administration in humans. It also highlights its potential as a therapeutic target with more effective treatments than what have been studied [3–5].

## Case presentation

**Fig. 1:**
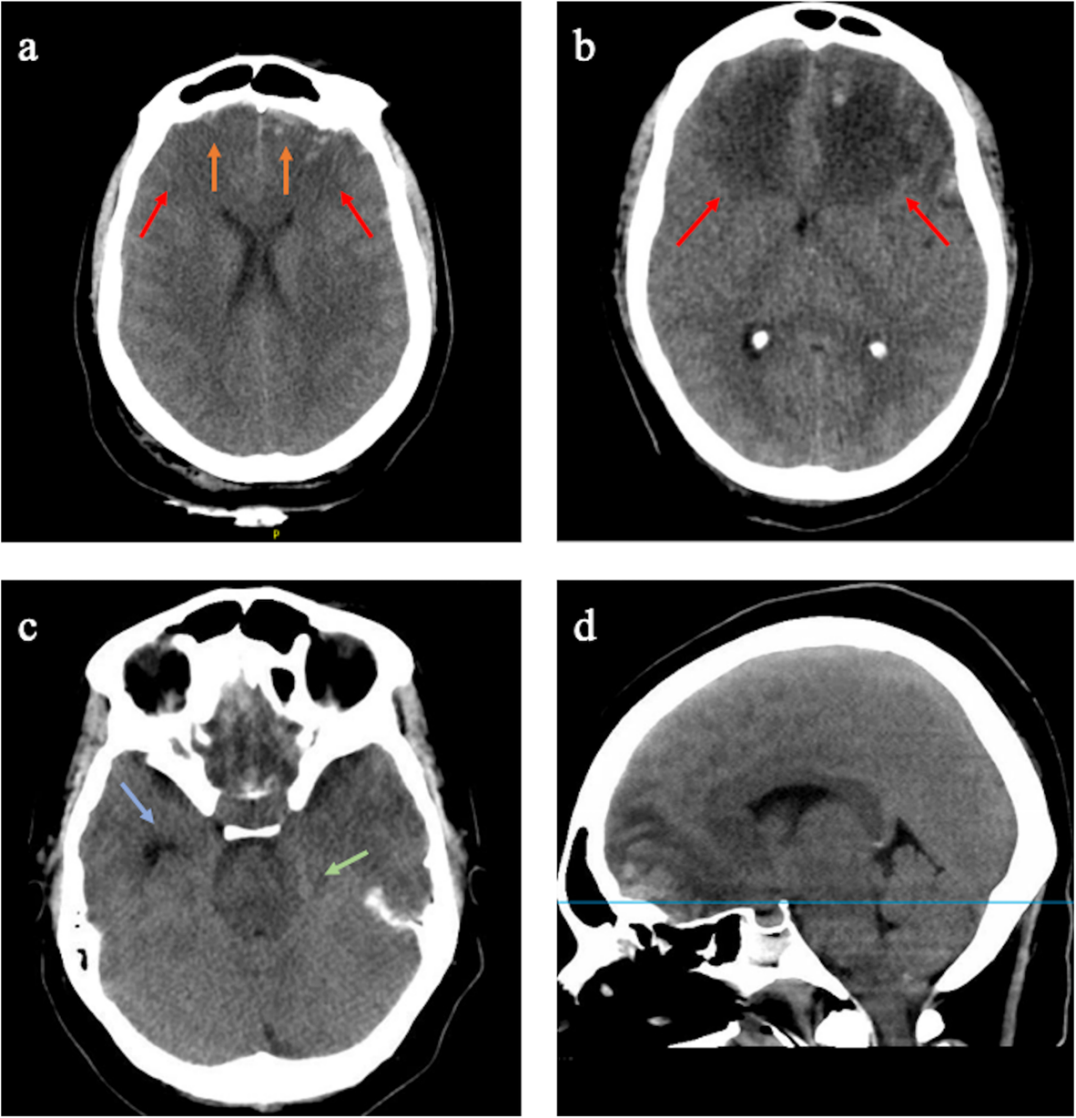
**a** Head CT on admission demonstrating bifrontal small subdural hematomas (orange arrows) and underlying bilateral hemorrhagic contusions (red arrows). **b** Head CT on day 4, shortly after IV iron administration, demonstrating significant expansion of perihematomal edema (PHE) (red arrows). **c**, **d** Head CT axial (at the level of midbrain) and sagittal planes, respectively, obtained immediately prior to EVD placement (approximately 12 h after iron administration)- notice compression of the ambient cistern (green arrow) and dilatation of the temporal horn of the right lateral ventricle (blue arrow)

A 46-year-old woman with no comorbidities was admitted to the intensive care unit (ICU) with acute TBI after she was struck, while jogging, by a motor vehicle traveling at 5 mph. Computed Tomography (CT) imaging revealed bifrontal small subdural hematomas with underlying hemorrhagic contusions, along with small subarachnoid hemorrhage (Fig. 1a). She was intubated upon arrival to the hospital and her Glasgow Coma Scale (GCS) quickly improved to 10 T. She was started on hypertonic saline 3% to achieve a target sodium goal of > 145 mmol/L, and a repeat head CT within 6 h showed stable hemorrhagic injuries and a small amount of PHE. The patient's sodium was maintained above 145 mmol/L and her condition appeared stable. Over the following two days she was extubated, and her GCS improved to 15. Her admission labs revealed microcytic (mean corpuscular volume (MCV) of 60.1 fL.) anemia with a hemoglobin of 7.8 g/dL (Ref range: 11.5–14.9 g/dL) which dropped to 5.7 g/dL on day 3 of hospital stay. She was transfused 2 units of packed red blood cells and extensive work up revealed no source of acute blood loss. Iron studies showed low serum iron of 15 mcg/dL (Ref range: 30–160 mcg/dL), and in the evening of day 3, she was given IV iron sucrose to replete her iron storages. Shortly after iron administration, her mental status deteriorated, and her left pupil became dilated and sluggish, indicating increased intracranial pressure (ICP). A head CT demonstrated significantly worsening bifrontal PHE (Fig. 1b), and she was given boluses of hypertonic saline and mannitol to which she initially responded clinically. However, 12 h later, her clinical condition deteriorated again with precipitous decline in GSC to 10, along with now bilaterally sluggish pupils. Follow-up head CT scans demonstrated increased PHE, with effacement of the ambient cistern (Fig. 1c, 1d) prompting intubation, maximizing hyperosmolar therapy and placement of an external ventricular drain (EVD) with a recorded ICP of 20 cm H2O after placement. EVD output was recorded to be 92 ml in the first 12 h following its placement. The patient received 3 doses of IV iron sucrose, but no further doses of iron were administered after it was suggested that iron administration might have been responsible for the worsening brain edema.

Between days 6 and 10 of hospital stay, initial attempts to wean hyperosmolar therapy demonstrated rebounding increase in ICP with correlating change in mental status warranting a slower wean. She was extubated on day 11 as her exam improved steadily, the EVD was removed on day 15, and her GCS remained stable at 14–15. The patient was discharged to a rehabilitation center after a 25-day hospital stay. She followed up with physical therapy and rehabilitation medicine where she continued to make progress in her recovery.

## Discussion and conclusions

Iron accumulates within brain parenchyma after ICH. Its ability to increase oxidative stress within cells has led to the belief that it plays a crucial role in secondary brain injury and therefore affects clinical outcome. Consequently, iron has been an appealing therapeutic target in the management of ICH. Deferoxamine, an iron-chelating agent, was shown to attenuate brain edema and neurological deficits in animal models [6]. However, clinical trials with deferoxamine were unsuccessful in showing benefit [3, 4]. Ran Lui et al. found that in human patients, iron overload after ICH was more prominent and lasted longer when compared to a rat model. This disparity may be due to differences in iron handling infrastructure between the rat and the human brain. It is possible that in order to achieve the neuroprotective effects seen in rat models, iron-chelating agents need to be administered to patients with an alternative regimen that may involve novel transport mechanisms through the blood brain barrier, expedited initiation or extended duration of treatment. Furthermore, several surgical trials have been done and many are still ongoing to study the effect of hematoma evacuation on PHE formation and clinical outcome in ICH [7–10].

Nevertheless, despite the iron chelator showing no difference in patient outcome in clinical trials, our patient’s malignant PHE progression could be a clinical demonstration of systemic iron's ability to cause brain injury. Administration of IV iron co-occurred with significant worsening of the patient’s mental status, and exponentially worsening PHE confirmed with serial CT imaging. (Fig. 1) Normally, an increase in systemic iron does not correlate with elevated iron levels within the central nervous system [1]. However, the patient received iron in the setting of blood brain barrier (BBB) disruption, permitting iron to slip past the damaged barrier. Additionally, iron affinity for brain tissue was increased due to acute inflammatory processes occurring in the setting of ICH. At the time of iron administration, the patient was in a perpetuated state of BBB disruption. Initial BBB disruption was a result of the patient’s head trauma and was sustained through the subsequent cytotoxic and vasogenic edema [11]. In such state, systemic iron was likely able to accumulate within the patient’s brain. Iron accumulation in brain tissue specifically may be a product of the brain’s extensive iron handling proteins. This infrastructure may confer a relatively high iron affinity to the brain relative to other tissues [1]. Despite this array of iron handling proteins, given an existing acute hemorrhage, the patient's iron handling systems were likely saturated even before IV iron was administered. This rendered the brain unable to effectively handle the excess iron, allowing significant oxidative stress to ensue. Importantly, the expansion of the patient’s edema occurred in the absence of confounding variables such as hyponatremia, hyperglycemia, or fever. Thus, the worsening edema and decline in mental status can be inferred to be a result from the increase in systemic iron.

The limitation of the findings in this case report is that it is a single instance in which iron administration was followed by worsening PHE. As such we lack sufficient statistical power to indicate causation over correlation. However, given the extensive evidence of iron toxicity to the brain, correlation of iron and brain edema, and lack of significant confounders, it is reasonable to indicate that the iron could have caused our patient’s worsening course.

The serial CT imaging showing the malignant course of the edema after iron was administered to our patient further potentiates our implied theory.

Healthcare providers should be aware of the potentially harmful effects of administering iron to a patient with sustained BBB disruption. During disruption, systemic iron can cross the barrier at liberty, enabling iron overload and increasing oxidative burden within the brain. Iron has been consistently shown to be toxic to the brain in preclinical studies, though clinical evidence is lacking for effective intervention. This case potentially demonstrates the ability of systemically administered iron to damage the brain in certain clinical settings. Eliminating iron from the brain or rendering it unable to form free radicals may still be a plausible therapeutic target in the management of ICH; pharmacological, surgical or both. We suggest further clinical research, potentially altering the administration regime or transport medium, selecting a drug with a different mechanism of action, or possibly combining iron-chelators with hematoma evacuation to rule out iron elimination’s potential as a therapeutic target in the management of ICH.

## Data Availability

Availability of data and materials is not applicable to this report.

## Abbreviations

PHE: Perihematomal edema
ICH: Intracerebral hemorrhage
TBI: Traumatic brain injury
CT: Computed tomography
EVD: External ventricular drain
IV: Intravenous
ICU: Intensive care unit
GCS: Glasgow Coma Scale
MCV: Mean corpuscular volume
ICP: Intracranial pressure
BBB: Blood brain barrier
